# Genome Sequences of the First Phages Infecting *Limnohabitans* Reveal Their Global Distribution and Metabolic Potential

**DOI:** 10.3390/microorganisms13061324

**Published:** 2025-06-06

**Authors:** Boxuan Deng, Raoqiong Che, Pinxin Zhu, Yongxia Wang, Zhiying Li, Shiying Zhang, Wei Xiao

**Affiliations:** 1 Yunnan Institute of Microbiology, School of Life Science, Yunnan University, Kunming 650500, China; dengboxuan52@gmail.com (B.D.); 15288131741@163.com (R.C.); zhupx@mail.ynu.edu.cn (P.Z.); wyx2321256@aliyun.com (Y.W.); zyli@ynu.edu.cn (Z.L.); 2 Yunnan Engineering Laboratory of Soil Fertility and Pollution Remediation, Yunnan Agricultural University, Kunming 650201, China

**Keywords:** *Limnohabitans*, bacteriophage, genome, distribution, auxiliary metabolic genes

## Abstract

Bacteriophages (phages) are one of the critical biotic drivers of prokaryotic community dynamics, functions, and evolution. Despite their importance in aquatic ecosystems, very few phages have been isolated from freshwater lakes, hampering our understanding of their ecological importance and usage in a variety of biotechnological applications. *Limnohabitans*, with a ubiquitous distribution, is a metabolically versatile, fast-growing, morphologically diverse freshwater lake bacterial genera. It is especially abundant in pH-neutral and alkaline aquatic habitats, where it represents an average of 12% of freshwater bacterioplankton and plays an important role in funneling carbon from primary producers to higher trophic levels. However, no phages infecting *Limnohabitans* have been reported to date. Here, we describe, for the first time, three phages infecting *Limnohabitans*, DC31, DC33, and YIMV22061, isolated from two freshwater lakes in China and characterized using genome content analysis and comparative genomics. DC31 and DC33, recovered from the eutrophic Dianchi Lake, with auxiliary metabolic genes (AMGs), associated with nucleotide metabolism, whereas YIMV22061, isolated from the oligotrophic Fuxian Lake, carried AMGs involved in antibiotic resistance. The AMGs they carried highlight their impacts on *Limnohabitans* in different environments. Comparative genomic analyses indicate that DC31, DC33, and YIMV22061 represent three novel species in the Caudoviricetes class. IMG/VR database alignment further reveal that these phages are widely distributed across diverse aquatic and terrestrial ecosystems globally, suggesting their ecological significance. This study provides a basis for better understanding *Limnohabitans*–phage interactions.

## 1. Introduction

Viruses are the most diverse, abundant, and ubiquitous biological entities in the biosphere, with an estimated global population of approximately 10^31^ [1,2], the majority of which are phages. It has become evident that phages play key roles in controlling microbial communities, promoting biogeochemical cycles, mediating horizontal gene transfer [3]. Freshwater lakes, as an integral part of the global hydrosphere, serve as nodes where various elements of the terrestrial surface system interact and are significant reservoirs of freshwater, flood mitigation, and genetic diversity. For more than two decades, metagenomic technologies have revealed the presence of novel and diverse bacteriophages (phages) in freshwater lakes and have significantly advanced our knowledge of viral diversity and potential functions [4,5,6]. Unfortunately, very few phages have been isolated from freshwater lakes thus far, such as the phages ɸFenriz, ɸHabibi, ɸMoody, and ɸVader (isolated from Lake Michigan) [7]; phages P19250A, P26059A, and P26059B (isolated from Lake Soyang) [8]; phages Lumi, Kuura, and Tiera (isolated from Lake Konnevesi) [9]; and cyanophages isolated from various freshwater lakes [10,11]. The sequencing and analysis of these phage genomes have deepened our understanding of the genomic diversity, evolution, and ecology of lake phages.

*Limnohabitans*, a genus within the Comamonadaceae family, has been observed in nearly every lake system worldwide in high relative abundance (9.4%) [12]. In our previous research, *Limnohabitans* was also the dominant genus in Dianchi Lake and Fuxian Lake [6]. In 2010, *Limnohabitans* was first isolated from Lake Mondsee in Austria. Since then, members of this genus have been isolated from freshwater systems in South Korea, Austria, the Czech Republic, and Brazil, and currently encompass six species (https://lpsn.dsmz.de/, accessed on 21 February 2025). Species of genus *Limnohabitans* play an important role in the transfer of carbon from primary producers to higher trophic levels and in facilitating energy flow, demonstrating significant ecological functions [13,14]. In addition to carbon sources, *Limnohabitans* exhibits high activity in the acquisition of glucose-6-phosphate and glycerol-3-phosphate, with a high proportion of strain-coded phosphate metabolism genes (e.g., *ugpQ*, *phoH*, *pstS*), indicating its significant role in phosphate utilization and cycling [15]. Of note, *Limnohabitans* has the potential for rapid response to environmental changes. Its composition and genomic characteristics are associated with variations in temperature and nutrient levels. The strain exhibits high growth rates and substrate uptake rates in eutrophic freshwater environments, allowing for it to quickly adapt to changes in nutrient availability [16]. Although *Limnohabitans* is a widely distributed and ecologically significant genus in freshwater ecosystems, to the best of our knowledge, no phages infecting *Limnohabitans* have been isolated to date.

Dianchi Lake, the sixth largest lake in China, is a typical eutrophic freshwater lake [17]. Approximately 20 km away, Fuxian Lake is the oligotrophic largest deep freshwater lake in China [18]. Our previous studies have revealed that *Limnohabitans* is the dominant genus in both the Dianchi and Fuxian Lakes [6]. In this study, we isolated *Limnohabitans* and their phages from these two lakes. Three *Limnohabitans* phages were isolated, and their genomes were analyzed to understand their taxonomic classification, auxiliary metabolic genes (AMGs), and distribution patterns. Our results reveal that the genomes of these three phages are distinct from those of other known phages, representing a group of novel, ecologically important, and globally distinctive phages. These results lay the groundwork to research the ecological functions of *Limnohabitans* phages and phage–*Limnohabitans* interactions.

## 2. Materials and Methods

### 2.1. Isolation of Limnohabitans and Phages

Surface water samples were collected from Dianchi Lake (24.87° N, 102.78° E) in December 2020 and from Fuxian Lake (24.63° N, 102.91° E) in April 2022. The samples were placed in sterile 25 L plastic containers and transported to the laboratory. *Limnohabitans* sp. strains were isolated from water samples using R2A media. The R2A medium used in this study was composed of (per liter) the following: 0.25 g tryptone, 0.5 g acid hydrolysate of casein, 0.5 g yeast extract, 0.5 g soluble starch, 0.3 g K_2_HPO_4_, 0.1 g MgSO_4_, 0.3 g sodium pyruvate, 0.25 g peptone, and 0.5 g glucose. The pH was adjusted to 7.2 before sterilization. For the solid medium, 15.0 g/L agar was added prior to autoclaving. The isolation of phages was performed according to our previously reported method [19]. Briefly, 10 mL of logarithmic-phase host culture was mixed with 30 mL of the water sample and incubated overnight at 32 °C, and shaking at 160 rpm. After incubation, the culture was centrifuged at 12,000 rpm for 20 min at 4 °C, and the supernatant was filtered through a 0.22 μm membrane (Millipore, Bedford, MA, USA). To isolate the phages, 1 mL of the filtered supernatant was mixed with 1 mL of logarithmic-phase host culture. After 20 min of adsorption at 32 °C, 5 mL of R2A semisolid medium (0.6% agar) was added, and the mixture was poured onto a preprepared solid agar plate. The plates were incubated and inverted at 32 °C until plaques appeared. After plaque formation, a single plaque was selected, along with the overlaying agar, and transferred into 1 mL of sterile water. The agar was thoroughly disrupted to release the phages and left to stand overnight at room temperature to allow for the complete diffusion of the phage particles into the water. On the following day, the suspension was centrifuged at 12,000 rpm for 5 min, and the supernatant was collected for serial dilution (from 10⁻^3^ to 10⁻^7^). For each dilution, 500 μL of the phage suspension was mixed with 500 μL of log-phase host bacteria and incubated at 32 °C for 20 min to allow for adsorption. The mixture was then added to 5 mL of R2A semi-solid medium (0.6% agar) and poured onto pre-prepared solid agar plates. After plaque formation, the purification steps were repeated multiple times until a single phage isolate was obtained.

### 2.2. Transmission Electron Microscopy (TEM)

To determine the morphology of the phages, the phage suspension was stained with 2% (*w*/*v*) sodium phosphotungstate for 1 min, air-dried, and then observed under a transmission electron microscope (JEM-2100; 200 kV, JEOL, Tokyo, Japan).

### 2.3. Extraction of Bacterial and Phage Genomic DNA

For the extraction of bacterial genomic DNA, a single colony was picked and added to 20 μL of lysis buffer (TakaRa, Beijing, China). The sample was lysed at 80 °C for 20 min, followed by centrifugation at 3000 rpm for 15 s. The supernatant was used as a PCR template.

The concentrated and purified phage suspension was filtered through a 0.22 μm pore size filter and then treated with DNase I and RNase A at 4 °C overnight. The DNase I- and RNase A-treated viral concentrate was inactivated by heating at 80 °C for 5 min [20]. Subsequently, proteinase K, EDTA (0.5 M), and 10% SDS were added, and the mixture was incubated at 55 °C for 3 h. Phage DNA was then extracted and purified by the phenol–chloroform method and ethanol precipitation [21].

### 2.4. Phylogenetic Analysis Based on the 16S rRNA Gene

The 16S rRNA gene was amplified using PCR using the universal primers 27F (5′-GCCCAGACTCCTACGGGA-3′) and 1492R (5′-TCTTCACACACGCGGCAT-3′), and then was sequenced. For the phylogenetic analysis, the best-fit evolutionary model was evaluated using ModelFinder [22] (version 1.5.4), and the maximum likelihood tree based on the 16S rRNA gene was performed using the IQ-TREE (version 1.6.12).

### 2.5. Phage Genome Sequencing and Bioinformatics Analysis

The genomic DNA libraries were constructed from the interrupted short DNA fragments and sequenced in paired-end mode by Guangdong Magigen Biotechnology Co., Ltd. (Guangzhou, China), using the Illumina NovaSeq platform. The raw sequencing data were filtered using SOAPnuke (v2.1.6) [23] and BWA (0.7.17) [24], and the filtered data were assembled with Megahit (1.2.9) [25]. Phage contigs were identified and assessed for genome completeness using CheckV (v2.9.0) [26].

Open reading frames (ORFs) were predicted using Prokka (1.14.6) [27]. Predictions for the functions of the phage ORFs were made using NCBI BLASTp in viral NR database-excluded uncultured sequences. The best-fit results are shown in Supplementary Figure S2 and Tables S1–S3. The annotation information for hypothetical proteins and ORFs without predicted information was matched against the Conserved Domain Database [28]. A complete phage genome map was generated using SnapGene (v6.0.2). The genome network analysis was conducted by combining PhaGCN2.2 [29], DIAMOND [30], and Cytoscape (v3.10.2) [31], using VirSorter2 to identify whether the phage genome was circular or linear. The phage genomes were aligned with the IMG/VR metagenomic database [32]; only high-quality results were used for subsequent analyses, and duplicate results were removed. Unless otherwise specified, the parameters for other software are the default values.

The amino acid sequences of major capsid proteins (MCPs) and terminase large subunits (TerLs) from phages were used to construct neighbor-joining phylogenetic trees with IQ-TREE. The phylogenetic tree was visualized using Chiplot (https://www.chiplot.online/, accessed on 1 April 2024) [33]. The phylogenetic tree based on the whole-genome sequences of phages was constructed using the VICTOR online server [34] with default parameters. ANI analysis was performed using the VIRIDIC, with all parameters set to default [35].

## 3. Results

### 3.1. Host Strains

The strains *Limnohabitans* sp. DCL3 and *Limnohabitans* sp. YIMB22184 were isolated from Dianchi Lake and Fuxian Lake, respectively. The phylogenetic tree based on 16S rRNA gene sequences showed that YIMB22184 is most closely linked to *L. planktonicus* II-D5, with a similarly of 99.17%. The strain closest to DCL3 was *L. parvus* II-B4, with a similarity of 99.05% (Supplementary Figure S1).

### 3.2. Biological Characteristics of Three Limnohabitans Phages

**Figure 1:**
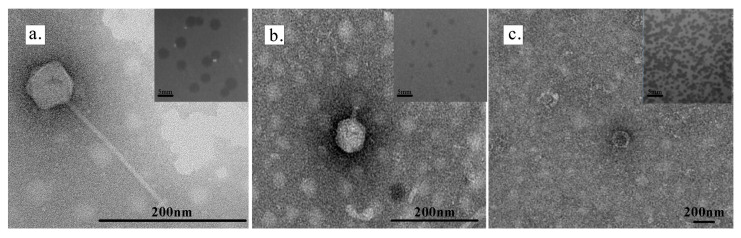
Transmission electron micrograph and plaque morphology of phages DC31 (**a**), DC33 (**b**), and YIMV22061 (**c**). The scale bars are indicated in the respective images.

Three phages—DC31, DC33, and YIMV22061—were isolated using two closely related bacterial hosts, *Limnohabitans* sp. DCL3 and *Limnohabitans* sp. YIMB22184. Phages DC31 and DC33 were isolated from Dianchi Lake using *Limnohabitans* sp. DCL3 as the host. After 24 h of cultivation, DC31 produced clear and round plaques with a diameter of 1.5–2 mm. TEM revealed that DC31 had an icosahedral head and a long tail, with a head diameter of 62.5 ± 5.5 nm and a tail length of 203 ± 3.4 nm (Figure 1a). After 48 h of cultivation, DC33 produced clear and round plaques with a diameter of 1–2 mm. TEM revealed that DC33 had a distinct tail with length of 32 ± 5 nm, and an icosahedral head with diameter of 74.5 ± 4.9 nm (Figure 1b).

When *Limnohabitans* sp. YIMB22184 was used as host, phage YIMV22061 was isolated from Fuxian Lake. After 24 h of cultivation, phage plaques with a diameter of 2.5–3 mm were observed. The center of the plaque was more transparent than the rest. No tail was observed for YIMV22061 by TEM, and its head had a diameter of 81 ± 9.9 nm (Figure 1c). Cross-infection tests revealed that phages DC33 and DC31 could infect YIMB22184, but phage YIMV22061 could not infect DCL3.

### 3.3. General Genomic Characteristics of Three Limnohabitans Phages

The genome of phage DC31 is a circular double-stranded DNA with a length of 76,209 bp and a G+C content of 37.58%. A total of 130 ORFs were predicted in the DC31 genome, including 6 tRNA genes. The total length of all the ORFs was 65,385 bp, accounting for 85.79% of the genome. Within this genome, 46 ORFs were assigned putative functions in terms of encoding structural proteins, lysis and assembly proteins, transcription and replication proteins, and metabolism and transcriptional regulators (Supplementary Figure S2A and Table S1). Additionally, 47 ORFs were annotated as hypothetical proteins, and 31 ORFs had no annotation information (Supplementary Figure S2A). A conserved domain analysis revealed that ORF56 may have a domain of the HU_IHF superfamily (E-value: 5.59 × 10^−7^, threshold: 0.01).

The genome of phage DC33 is a circular double-stranded DNA with a length of 39,048 bp and a G+C content of 54.26%. In the DC33 genome, 52 ORFs were predicted and accounted for 94.73% of the total genome length. Within this genome, 16 ORFs were assigned putative functions in terms of encoding structural proteins, lysis and assembly proteins, transcription and replication proteins, and metabolism (Supplementary Figure S2B and Table S2). Thirty-two ORFs were annotated as hypothetical proteins, whereas four ORFs had no annotation information (Supplementary Figure S2B). Conserved domain analysis revealed that ORF7 may have a domain of the N-acyltransferase superfamily (E-value: 1.53 × 10^−11^, threshold: 0.01), which is involved in catalyzing acyl transfer to substrates and is related to bacterial antibiotic resistance [36]. Four ORFs are predicted to contain a single domain, including a HTH_XRE (ORF10), UvrA protein (ORF29), Golgin_A5 domain (ORF39), and PHA00658 domain (ORF39).

The YIMV22061 genome is a circular double-stranded DNA with a length of 40,749 bp and a G+C content of 64.76%. The genome is predicted to contain 50 ORFs, including 1 tRNA, and account for 90.94% of the total genome length. Twenty-five ORFs were assigned putative functions in terms of encoding structural proteins, lysis and assembly proteins, transcription and replication proteins, and metabolism and integrase. Additionally, 16 ORFs were annotated as hypothetical proteins, and 9 ORFs had no annotation information (Supplementary Figure S2C and Table S3).

In the whole genomes of DC31, DC33, and YIMV22061, the MCP are encoded by ORF126, ORF52, and ORF38, respectively. The MCP is the primary component that construct the phage capsid. In DC31, both ORF4 and ORF5 encode tail proteins, whereas in DC33, the tail protein is encoded by ORF42. Although the phage YIMV22061 contains a tail protein-encoding gene, ORF36, unfortunately, no distinct tail structure was observed using TEM.

The head-to-tail connecting protein (ORF5) and the portal protein (ORF42) were annotated in phages DC33 and YIMV22061, respectively. These two proteins share similar functions, both of which are involved in the structural assembly of the phage. The primary role of these proteins is to connect and assemble the DNA-filled capsid and the tail of the phage through separate assembly pathways [37].

The TerL was annotated in DC31 (ORF12), DC33 (ORF9), and YIMV22061 (ORF25). TerL is typically responsible for binding to the prohead, DNA translocation and cleavage, and ATP binding during the packaging process of the phage genome. This large subunit, in conjunction with the small subunit that is responsible for DNA recognition and binding, forms the terminase complex.

Some double-stranded DNA phages produce a soluble cell-wall-degrading enzyme known as endolysin [38]. Endolysins disrupt the cell by degrading the links that are crucial for the integrity of peptidoglycan [39]. We annotated endolysins in DC31 (ORF46), DC33 (ORF38), and YIMV22061 (ORF9). We also annotated the presence of a Holin protein (ORF39) in the genome of YIMV22061, emphasizing the phage’s lytic capacity.

### 3.4. Metabolic Potential of Three Limnohabitans Phages

Through functional annotation of the protein groups, we identified several AMGs from these *Limnohabitans* phage genomes. In DC31, ORF22 is predicted to encode the PhoH family protein. PhoH is related to the regulation of phosphorus metabolism and is typically associated with the bacterial Pho regulon (phosphate response regulon), which governs the assimilation of phosphate sources to promote bacterial growth [40]. ORF40 encodes a WYL domain-containing protein. The protein regulates gene expression in response to nucleic acid signals, helping the cell cope with environmental stresses such as DNA damage [41]. ORF42 encodes phosphoglycerate kinase. This enzyme catalyzes the transfer of a phosphate group from 1,3-bisphosphoglycerate to Mg-ADP, resulting in the formation of 3-phosphoglycerate and Mg-ATP during glycolysis. This enzyme is highly conserved across biological systems and is considered an essential enzyme in many organisms [42]. It is involved in glycolytic reactions and is related to energy metabolism [43]. ORF71 encodes dCMP deaminase, which plays a crucial role in DNA synthesis and nucleotide metabolism [44]. ORF109 encodes thioredoxin, a protein required for DNA replication [45], and plays a key role in reducing the level of intracellular protein disulfides [46]. ORF110 was homologous with D-alanyl-D-alanine carboxypeptidase, which is a primary target for β-lactam antibiotics [47].

In DC33, ORF8 was annotated as aspartyl/asparaginyl beta-hydroxylase, which catalyzes the β-hydroxylation of specific aspartic acid or asparagine residues in proteins [48]. ORF23 encodes a mazG-like family protein. *mazG* is typically classified as an NTPase, a member of the nucleotide triphosphatase family, which hydrolyses nucleoside triphosphates (such as ATP or GTP) [49]. ORF35 encodes the RecT recombinational DNA repair protein, a key DNA recombination repair protein and a member of the DNA single-strand annealing protein family. It plays a role in both the RecA-dependent and RecA-independent DNA recombination pathways and is widely present in bacteria [50].

In YIMV22061, ORF2 hit GCN5-related N-acetyltransferase with identity of 46%. The first described acetyltransferase is bacterial aminoglycoside acetyltransferase, which was shown to confer antibiotic resistance. The importance of this ubiquitous modification has become progressively established in recent decades, and it is now known to involve in processes ranging from protein synthesis and gene expression to detoxification and virulence [51]. ORF18 encodes MarR transcriptional regulators, which primarily regulate gene expression by binding to specific DNA sequences and related to antibiotic resistance, stress response, and metabolic regulation [52,53,54].

### 3.5. Globally Distribution of Three Limnohabitans Phages

To assess the biogeographical distribution of *Limnohabitans* phages, we performed a viromic read-mapping analysis. The results show that the genomes of DC31, DC33, and YIMV22061 were highly similar to 36, 14, and 92 sequences in the IMG/VR datasets, respectively.

DC31-like phages are widely distributed across multiple ecosystems in North America (United States, Canada), Southeast Asia (Singapore), and Antarctica, including freshwater lakes, seawater, river shale gas environments, and wastewater treatment systems. In contrast, DC33-like phages exhibit a more localized geographic distribution, primarily found in North America (United States, Canada) and Europe (Switzerland) within freshwater lakes, rivers, and mine pit ponds. YIMV22061 demonstrates the broadest distribution, occurring in North America (United States, Canada), Europe (Sweden, Spain), Asia (China, Japan), Africa (South Africa, Congo), and Antarctica, and inhabits various environments such as freshwater wetland sediments, root nodules, the rhizosphere soil of graminaceous plants, bioreactor wood chips, subway systems, peat permafrost, marine ecosystems, and wastewater systems.

### 3.6. The First Genome of Phages Infecting Limnohabitans

Using BLASTn from the NR database, we searched against the genomes of DC31, DC33, and YIMV22061 and those of other phages. The results indicate that DC31 has the highest homology with *Curvibacter* phage P26059A (KY981271), with a similarity of 75.58%, but the coverage was only 1%. The whole genome of phage DC33 is most similar to that of phage ctQRb9 (MW202722, isolated from Manatee Spring, USA), with a similarity of 71.31% and coverage of 7%. For YIMV22061, a subsequent comparison revealed that its genome is closest to that of the *Ralstonia* phage P-PSG-11-1 (MN270890), with a similarity of 73.20% and coverage of 4%.

**Figure 2:**
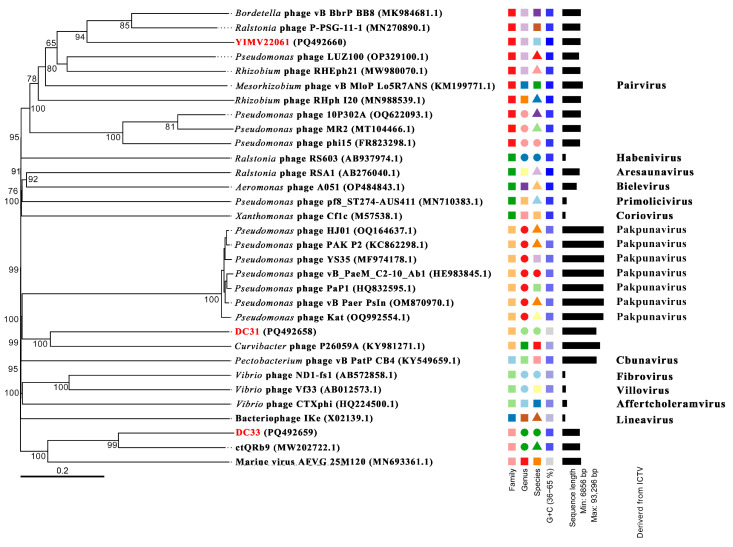
Phylogenetic analysis of the genome sequences of DC31, DC33, and YIMV22061 using VICTOR. The classifications of families and genera are derived from the VICTOR evaluation results. Genus names were derived from ICTV. The numbers next to the nodes represent the supporting values, which indicate the consistency or reliability of the corresponding branches in the phylogenetic analysis. Higher values reflect greater confidence in the validity of the branch. The red text emphasizes the three phages we isolated. Viruses with no genus name indicate that ICTV is not currently publishing their classification status.

Additionally, a total of 29 top-matching viral genomes were selected using BLAST (https://blast.ncbi.nlm.nih.gov/Blast.cgi, accessed on 20 March 2025) from NCBI and a subsequent phylogenetic analysis. A whole-genome phylogenetic tree was constructed, and this indicated that DC31 and DC33 clustered together in a large cluster, suggesting a relatively closer relationship between these two phages (Figure 2). Phage DC31 clustered with *Curvibacter* (Comamonadaceae) phage P26059A, while phage DC33 clustered with phage ctQRb9. These results are consistent with the results from BLASTn. *Ralstonia* phage P-PSG-11-1, which had the highest identity with YIMV22061 according to BLASTn, was located in another small cluster, showing a comparatively distant phylogenetic relationship.

**Figure 3:**
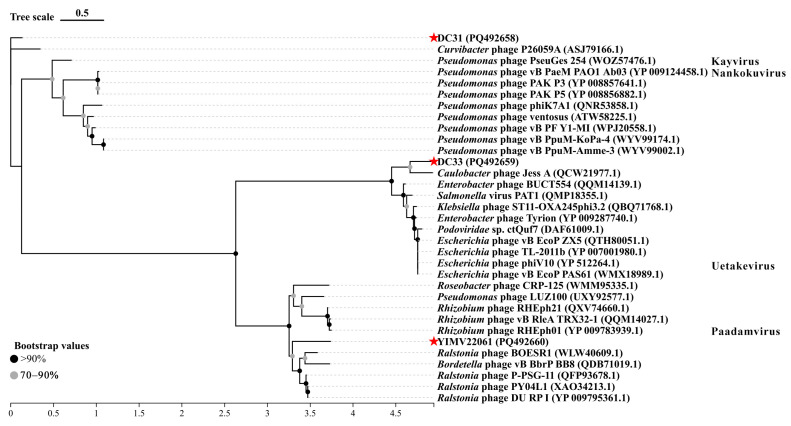
Phylogenetic analysis of phages DC31, DC33, and YIMV22061 on the basis of the major capsid protein amino acid sequence. Bootstrap values represent the support for branches assessed by resampling; higher values indicate more reliable evolutionary relationships. The red stars emphasizes the three phages we isolated. Phylogenetic trees were constructed using the maximum likelihood method in IQ-TREE. All the parameters are default.

Moreover, phylogenetic trees were established based on the MCP and TerL using 30 best hit sequences. In the MCP tree, DC31 clustered with *Curvibacter* phage P26059A, which is consistent with the results from the whole-genome phylogenetic tree. DC33 is closely related to *Caulobacter* phage Jess A (QCW21977), forming a distinct clade with longer branch lengths, while YIMV22061 formed separate clusters with other phages with longer branch lengths, indicating greater evolutionary divergence (Figure 3).

**Figure 4:**
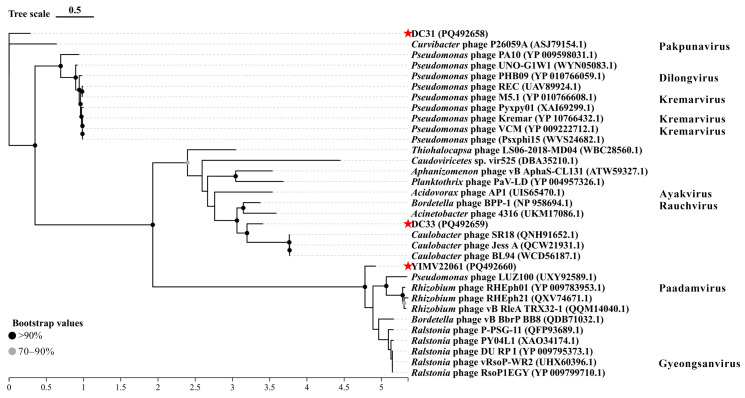
Phylogenetic analysis of phages DC31, DC33, and YIMV22061 on the basis of the terminase large subunit amino acid sequence. Bootstrap values represent the support for branches assessed by resampling; higher values indicate more reliable evolutionary relationships. The red stars emphasizes the three phages we isolated. Phylogenetic trees were constructed via the maximum likelihood method in IQ-TREE. All the parameters are default.

The results of the phylogenetic analysis of TerL indicate that DC31 clusters with *Curvibacter* phage P26059A, which is consistent with the results from the whole-genome and MCP phylogenetic tree. In contrast, DC33 and YIMV22061 exhibit greater genetic distances from other phages, similar to the results of the MCP phylogenetic analysis (Figure 4).

**Figure 5:**
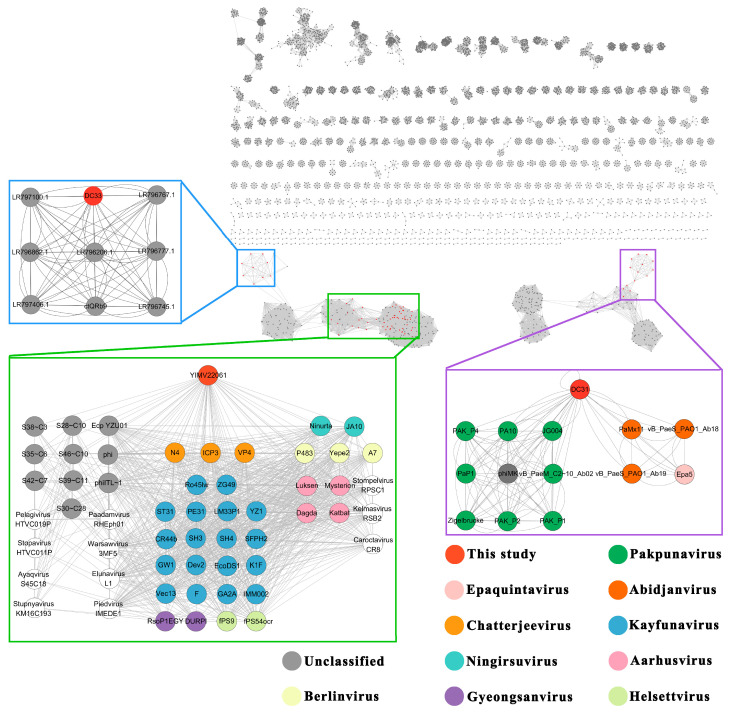
Genomic network diagram. Genomic network analysis was performed using PhaGCN2.2 and Cytoscape. The analysis utilized an in-house database (last updated in February 2023) and included 14 best-hit sequences to DC33 from GenBank. Different colored circles represent different viral genera. White circles represent unique viral genera and are labeled with the genus name. Edges indicate correlations between viral sequences, and virus clusters are shown as differently colored boxes. The classification status of the viruses is derived from the ICTV.

To further elucidate the taxonomy of DC31, DC33, and YIMV22061, a genome network analysis were performed. The results reveal that 13 viruses are directly associated with DC31, which belong to Pakpunavirus, Epaquintavirus, and Abidjanvirus (all belong to Caudoviricetes). For DC33, there are eight viruses with direct associations, but none of these have a clearly defined taxonomic status in the ICTV. In the case of YIMV22061, 55 viruses are directly linked to it, such as Kayfunavirus, Chatterjeevirus, Berlinvirus, and Kayfunavirus, which belong to Studiervirinae (Figure 5).

**Figure 6:**
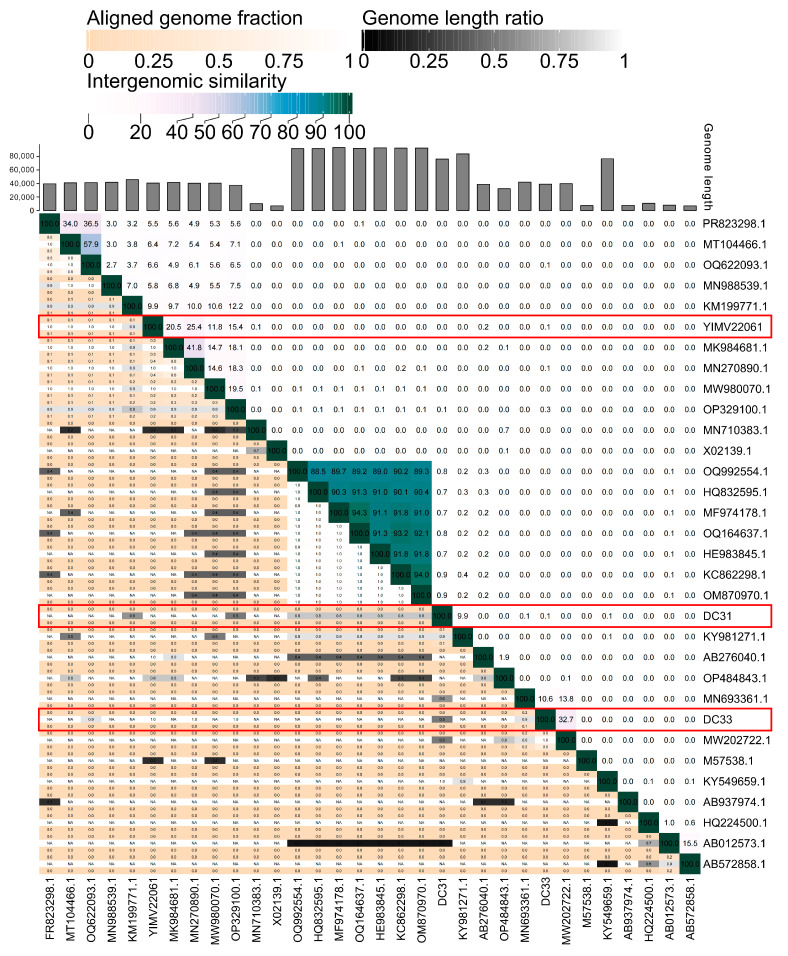
ANI analysis of the genome sequences of DC31, DC33, and YIMV22061 was performed using VIRIDIC. The larger the number in the upper right triangle of the matrix, the higher the correlation. “NA” indicates that ANI values are not available for these comparisons. The red box emphasizes the three phages we isolated.

The ANI analysis of these 29 phages included in Figure 2 shows a significant distance between the DC31, DC33, and YIMV22061 and other known viruses. DC31 was most similar to the *Curvibacter* phage P26059A, with an intergenomic similarity of 9.9%. The highest value between DC33 and ctQRb9 was only 32.7%. YIMV22061 was most similar to *Ralstonia* phage P-PSG-11-1, with an intergenomic similarity of 25.4% (Figure 6). Overall, the above evidence suggests that these three phages represent novel viral groups in the Caudoviricetes class.

## 4. Discussion

The importance of the *Limnohabitans* genus for freshwater food webs has been proposed to be equivalent to that of the SAR11 taxon for marine food webs [13], while phages can interact with *Limnohabitans* and strongly impact community compositions, and thus affect the metabolism of the host. The present study focuses on the analysis of three novel phages infecting *Limnohabitans*. This research aims to characterize the genome sequence and investigate the distribution and metabolic potential of these phages. The results contribute to our understanding of phage–*Limnohabitans* interactions.

The *Limnohabitans* phages were successfully isolated from two lakes in China. The morphological characterization using TEM revealed that DC31 and DC33 belongs to a head-tailed phage (Figure 1). Despite many attempts, the tail of YIMV22061 was not observed using TEM, which disagrees with the genomic analysis (Supplementary Figure S2C and Table S3). This might be because the tail of YIMV22061 is very fragile and prone to detachment during the phage collection process. The host range analysis demonstrated that DC31 and DC33 exhibited strong lytic activity against *Limnohabitans* sp. DCL3 and YIMB22184, but YIMV22061 infect YIMB22184 only. The host range is influenced not only by the phage’s and the host bacterium’s genetic makeup and physical structure, but also by the environment where they interact [55]. Although DC31 and DC33 share the same host range, their tail proteins are not identified (Supplementary Tables S1 and S2), indicating that they may recognize the host through different receptors. Furthermore, the identification of specific genes associated with DNA modification within the DC31 and DC33 genome contribute to the phage’s ability to evolve and overcome bacterial defense mechanisms, which broaden the host range.

Viral infection can reprogram the host metabolism in multiple pathways through the expression of AMGs. The *phoH* gene encoded by DC31 has been found in previous studies and is widely distributed in viruses from various environments [56,57]. The phage-encoded *phoH* may play a role in counteracting host stress responses triggered by phage infection and in enhancing the yield of newly produced phage particles [58]. Under phosphorus-limited conditions, the expression of *phoH* varies among different bacteria. For example, *Escherichia coli* and *Corynebacterium glutamicum* induce the expression of this gene under phosphate starvation [59], whereas *phoH* in *Prochlorococcus* strains is not induced under phosphate limitation [60]. However, bioinformatic analysis shows that *phoH* could also be involved in phospholipid metabolism and RNA modification or fatty acid beta-oxidation [61].

Our results reveal that DC31 encodes a dCMP deaminase (ORF71), but its host DCL3 does not. dCMP deaminase plays a key role in DNA synthesis and nucleotide metabolism by catalyzing the conversion of dCMP to dUMP. dUMP is the direct precursor for dTMP synthesis, which is essential for DNA synthesis [62]. We found that the host strain DCL3 has a G+C content of 59.25%, which is greater than that of phage DC31 (37.58%). The significant difference in G+C content between the host and the phage may pose challenges for the synthesis of the progeny phage [63]. Previous radioactive tracing experiments on marine phage‒host interactions have indicated that the nucleotides in phage DNA primarily originate from host nucleotides [64], suggesting that host resources could influence viral outbreaks [65]. Therefore, the dCMP deaminase may help phage DC31 synthesize their own DNA using host nucleic acid resources.

In the DC33 genome, a *mazG* gene (ORF23) related to phosphorus metabolism was annotated. *mazG* is involved in the nucleic acid metabolism and catalyzes the hydrolysis of nucleoside triphosphates (NTPs) to produce nucleoside monophosphates (NMPs) and pyrophosphate (PPi) [49]. Previous studies have shown that the substrate specificity of the *mazG* gene encoded by viruses allows for it to preferentially hydrolyse dGTP and dCTP deoxynucleotides from the genome of the high-GC-content host *Synechococcus*, thereby facilitating their recycling and ultimately promoting the replication of the AT-rich phage genome [66]. Considering that the G+C content of the host strain DCL3 is 59.25% and that of the phage DC33 is 54.26%, which is lower than that of the host, ORF23 may assist in the replication process of DC33. Other studies have shown that *mazG*, encoded by cyanophage, may induce the host to mimic a nutrient-rich cellular state, thus optimizing host cell physiology to promote macromolecule synthesis and virus replication. Further research has shown that in Mycobacterium tuberculosis lacking *mazG*, DNA instability occurs, indicating that this gene plays a role in genome protection and antioxidation [67]. *mazG* encoded by phages can deplete (p)ppGpp in the host cell, thereby blocking the host cell’s suicide modules (such as mazEF) activated by (p)ppGpp. The depletion of this metabolite weakens the host immune response, enabling the phage to replicate and spread more efficiently within the host cell [68]. The widespread presence of *mazG* in phages may indicate that it provides a selective advantage, playing a crucial role in the survival and propagation of the phage.

Unlike phages DC31 and DC33, which encode AMGs related to nucleotide metabolism, YIMV22061 encodes AMGs related to antibiotic resistance, such as the GNAT family N-acetyltransferase (ORF85) and *marR* transcriptional regulators (ORF18). This could be due to the phage’s habitat. DC31 and DC33 were isolated from eutrophic lakes. Nucleotide metabolism-related AMGs may aid the host in more efficiently utilizing phosphorus from the eutrophic habitat, ultimately increasing the number of phage progeny or enhancing the competitive advantage of the host. In contrast, phage YIMV22061 was isolated from the oligotrophic lake. The AMGs encoded by YIMV22061 are linked to antibiotic resistance. Phages carrying these AMGs may confer a population-level advantage to their hosts in intense interspecies competition [69]. But this hypothesis needs to be analyzed in more phages.

The biogeographical distribution of DC31-, DC33-, and YIMV22061-like phages showed that these phages can be detected globally, from terrestrial to marine regions, as well as from tropical to polar regions. DC31- and DC33-like phages are commonly distribution in aquatic habitats, and their distribution ranges from temperate to cold regions, while YIMV22061-like phages exhibit a broader global distribution and are detected in diverse habitats, such as soil, sediment, wastewater, and marine environments. It spans diverse climatic zones, including temperate, subarctic, tropical, and polar regions. These results suggest that, on the one hand, these phages may have a broad host range, and, on the other hand, their hosts can adapt to diverse environments, which may benefit from the protection of the hosts by the phage’s AMGs, such as antibiotic resistance genes encoded by YIMV22061. The wide distribution of *Limnohabitans* phage is in agreement with the general distribution of *Limnohabitans* in different environment. The widespread presence of these phages across different climatic zones suggests that their hosts may be broadly distributed and capable of adapting to varying temperatures and salinity. Props and Denef found that thermal adaptation may be a more important factor in the overall microdiversification within the *Limnohabitans* genus [13]. A broader isolation of *Limnohabitans* phages across diverse geographic regions and freshwater systems will be essential to fully elucidate their ecological diversity and host interactions.

Phages exhibit huge genomic diversity, and its classification faces many challenges. Comprehensive comparative and evolutionary analyses are necessary to better understand the diversity and modular evolution of phages. Such analyses go beyond single-gene phylogenies and can reveal conserved genomic structures, improving insights into phage classification and their ecological roles. However, it must be acknowledged that, currently, regardless of the phylogenetic tree used, the accurate classification of phages remains unattainable. In this study, the BLASTn shown that DC31, DC33, and YIMV22061 have the highest identity, being >70%, but a coverage of 1–7%. Furthermore, phylogenetic analyses based on genome and single genes indicate that all three phages are quite distinct from other known viruses (Figure 2, Figure 3 and Figure 4). Moreover, genomic network analyses revealed that these three phages were not classified into any of the virus clusters or were associated with unclassified viruses (Figure 5). In addition, the ANI analyses show that the intergenomic similarities between all three phages and all similar phages were less than 35% (Figure 6), which is lower than the current criterion for defining a new genus and species of bacteria and archaeal viruses (which requires genome identities of 70% and 95%, respectively). All of these genomic results and morphologies supported that phages DC31, DC33, and YIMV22061 were novel species of the Caudoviricetes class.

## 5. Conclusions

Here, we isolated and report genomes of the first *Limnohabitans* phages. These phages represent three novel groups in the Caudoviricetes class and are distributed in diverse habitat worldwide. These phages encode various AMGs, which may reprogram the host’s metabolism, especially the nucleotide metabolism and antibiotic resistance. Our work expands current knowledge regarding the diversity and evolution of lake phages, highlighting the ecological function of *Limnohabitans* phages. The *Limnohabitans* phages we isolated provide a unique model system for studying lake virus–host interactions.

## Data Availability

All the data generated or analyzed in this study are included in this published article and the Supplementary Materials. The following genomic data for phages DC31, DC33, and YIMV22061 and strains *Limnohabitans* sp. DCL3 and *Limnohabitans* sp. YIMB22184 are available in the NCBI database: PQ492658, PQ492659, PQ492660, CP171842, CP171843.

## Supplementary Materials

The following supporting information can be downloaded at: https://www.mdpi.com/article/10.3390/microorganisms13061324/s1, Figure S1: The best evolutionary model for the sequence was evaluated using ModelFinder. phylogenetic tree was constructed using this model via maximum likelihood method on the IQ-TREE website, with a bootstrap value of 1000, and all other options were set to default. Graphical enhancement was performed via the online tool Chiplot (https://www.chiplot.online/, accessed on 1 April 2024); Figure S2: Genome maps of DC31 (A), DC33 (B), and YIMV22061 (C). Arrows represent ORFs, with different colors indicating different functions. Green: structural proteins; blue: lysis and assembly genes; pink: transcription and replication genes; red: auxiliary metabolism genes; gold: modification genes; purple: integrase gene; Table S1: Phage DC31 ORFs annotation information (Only display genes with functional annotations); Table S2: Phage DC33 ORFs annotation information (Only display genes with functional annotations); Table S3: Phage YIMV22061 ORFs annotation information (Only display genes with functional annotations).

## Abbreviations

The following abbreviations are used in this manuscript:

AMG	Auxiliary metabolic gene
ORF	Open reading frame
ATP	Adenosine triphosphate
dCMP	Deoxycytidine monophosphate
dUMP	Deoxyuridine monophosphate
dTMP	Deoxythymidine monophosphate
MCP	Major capsid protein
TerL	Terminase large subunit

## References

[1] Sime-Ngando T. (2014). Environmental bacteriophages: Viruses of microbes in aquatic ecosystems. Front. Microbiol..

[2] Cobián Güemes A.G., Youle M., Cantú V.A., Felts B., Nulton J., Rohwer F. (2016). Viruses as winners in the game of life. Annu. Rev. Virol..

[3] Suttle C.A. (2007). Marine viruses—Major players in the global ecosystem. Nat. Rev. Microbiol..

[4] Kavagutti V.S., Andrei A.Ş., Mehrshad M., Salcher M.M., Ghai R. (2019). Phage-centric ecological interactions in aquatic ecosystems revealed through ultradeep metagenomics. Microbiome.

[5] Zhao A., Lu Y., Li Q., Li T., Zhao J. (2023). Metagenomics reveals the diversity and role of surface-water microbes in biogeochemical cycles in lakes at different terrain ladders. Front. Environ. Sci..

[6] Che R., Bai M., Xiao W., Zhang S., Wang Y., Cui X. (2022). Nutrient levels and prokaryotes affect viral communities in plateau lakes. Sci. Total. Environ..

[7] Malki K., Kula A., Bruder K., Sible E., Hatzopoulos T., Steidel S., Watkins S.C., Putonti C. (2015). Bacteriophages isolated from Lake Michigan demonstrate broad host-range across several bacterial phyla. Virol. J..

[8] Moon K., Kang I., Kim S., Kim S.J., Cho J.C. (2018). Genomic and ecological study of two distinctive freshwater bacteriophages infecting a *Comamonadaceae* bacterium. Sci. Rep..

[9] Laanto E., Oksanen H.M. (2023). Three phages from a boreal lake during ice cover infecting *Xylophilus*, *Caulobacter*, and *Polaromonas* species. Viruses.

[10] Yang F., Jin H., Wang X.Q., Li Q., Zhang J.T., Cui N., Jiang Y.L., Chen Y., Wu Q.F., Zhou C.Z. (2020). Genomic analysis of Mic1 reveals a novel freshwater long-tailed cyanophage. Front. Microbiol..

[11] Zhang D., He Y., Gin K.Y. (2022). Genomic characterization of a novel freshwater cyanophage reveals a new lineage of cyanopodovirus. Front. Microbiol..

[12] Simek K., Kasalicky V., Jezbera J., Jezberová J., Hejzlar J., Hahn M.W. (2010). Broad habitat range of the phylogenetically narrow R-BT065 cluster, representing a core group of the Betaproteobacterial genus *Limnohabitans*. Appl. Environ. Microbiol..

[13] Props R., Denef V.J. (2020). Temperature and nutrient levels correspond with lineage-specific microdiversification in the ubiquitous and abundant freshwater genus *Limnohabitans*. Appl. Environ. Microbiol..

[14] Kasalický V., Zeng Y., Piwosz K., Šimek K., Kratochvilová H., Koblížek M. (2018). Aerobic anoxygenic photosynthesis is commonly present within the genus *Limnohabitans*. Appl. Environ. Microbiol..

[15] Rofner C., Sommaruga R., Pérez M.T. (2016). Differential utilization patterns of dissolved organic phosphorus compounds by heterotrophic bacteria in two mountain lakes. FEMS Microbiol. Ecol..

[16] Šimek K., Kasalický V., Zapomělová E., Horňák K. (2011). Alga-derived substrates select for distinct betaproteobacterial lineages and contribute to niche separation in *Limnohabitans* strains. Appl. Environ. Microbiol..

[17] He J., Wu X., Zhang Y., Zheng B., Meng D., Zhou H., Lu L., Deng W., Shao Z., Qin Y. (2020). Management of water quality targets based on river-lake water quality response relationships for lake basins–a case study of Dianchi Lake. Environ. Res..

[18] Lin Z., Peng S. (2022). Comparison of multimodel simulations of land use and land cover change considering integrated constraints-A case study of the Fuxian Lake basin. Ecol. Indic..

[19] Fu C.Q., Zhao Q., Li Z.Y., Wang Y.X., Zhang S.Y., Lai Y.H., Xiao W., Cui X.L. (2016). A novel Halomonas ventosae-specific virulent halovirus isolated from the Qiaohou salt mine in Yunnan, Southwest China. Extremophiles.

[20] Diao K., Li G., Sun X., Yi H., Zhang S., Xiao W. (2023). Genomic Characterization of a Halovirus Representing a Novel Siphoviral Cluster. Viruses.

[21] Lekunberri I., Villagrasa M., Balcázar J.L., Borrego C.M. (2017). Contribution of bacteriophage and plasmid DNA to the mobilization of antibiotic resistance genes in a river receiving treated wastewater discharges. Sci. Total. Environ..

[22] Kalyaanamoorthy S., Minh B.Q., Wong T.K.F., von Haeseler A., Jermiin L.S. (2017). ModelFinder: Fast model selection for accurate phylogenetic estimates. Nat. Methods.

[23] Chen Y., Chen Y., Shi C., Huang Z., Zhang Y., Li S., Li Y., Ye J., Yu C., Li Z. (2018). SOAPnuke: A MapReduce acceleration-supported software for integrated quality control and preprocessing of high-throughput sequencing data. Gigascience.

[24] Li H., Durbin R. (2009). Fast and accurate short read alignment with Burrows–Wheeler transform. Bioinformatics.

[25] Li D., Liu C.M., Luo R., Sadakane K., Lam T.W. (2015). MEGAHIT: An ultrafast single-node solution for large and complex metagenomics assembly via succinct de Bruijn graph. Bioinformatics.

[26] Nayfach S., Camargo A.P., Schulz F., Eloe-Fadrosh E., Roux S., Kyrpides N.C. (2021). CheckV assesses the quality and completeness of metagenome-assembled viral genomes. Nat. Biotechnol..

[27] Seemann T. (2014). Prokka: Rapid prokaryotic genome annotation. Bioinformatics.

[28] Marchler-Bauer A., Panchenko A.R., Shoemaker B.A., Thiessen P.A., Geer L.Y., Bryant S.H. (2002). CDD: A database of conserved domain alignments with links to domain three-dimensional structure. Nucleic Acids Res..

[29] Jiang J.Z., Yuan W.G., Shang J., Shi Y.H., Yang L.L., Liu M., Zhu P., Jin T., Sun Y., Yuan L.H. (2023). Virus classification for viral genomic fragments using PhaGCN2. Brief. Bioinform..

[30] Buchfink B., Ashkenazy H., Reuter K., Kennedy J.A., Drost H.G. (2023). Sensitive clustering of protein sequences at tree-of-life scale using DIAMOND DeepClust. BioRxiv.

[31] Shannon P., Markiel A., Ozier O., Baliga N.S., Wang J.T., Ramage D., Amin N., Schwikowski B., Ideker T. (2003). Cytoscape: A software environment for integrated models of biomolecular interaction networks. Genome Res..

[32] Camargo A.P., Nayfach S., Chen I.A., Palaniappan K., Ratner A., Chu K., Ritter S.J., Reddy T.B.K., Mukherjee S., Schulz F. (2023). IMG/VR v4: An expanded database of uncultivated virus genomes within a framework of extensive functional, taxonomic, and ecological metadata. Nucleic Acids Res..

[33] Xie J., Chen Y., Cai G., Cai R., Hu Z., Wang H. (2023). Tree Visualization By One Table (tvBOT): A web application for visualizing, modifying and annotating phylogenetic trees. Nucleic Acids Res..

[34] Meier-Kolthoff J.P., Göker M. (2017). VICTOR: Genome-based phylogeny and classification of prokaryotic viruses. Bioinformatics.

[35] Moraru C., Varsani A., Kropinski A.M. (2020). VIRIDIC–a novel tool to calculate the intergenomic similarities of prokaryote-infecting viruses. Viruses.

[36] Okamoto S., Suzuki Y. (1965). Chloramphenicol-, dihydrostreptomycin-, and kanamycin-inactivating enzymes from multiple drug-resistant *Escherichia coli* carrying episome‘R’. Nature.

[37] Lurz R., Orlova E.V., Günther D., Dube P., Dröge A., Weise F., van Heel M., Tavares P. (2001). Structural organization of the head-to-tail interface of a bacterial virus. J. Mol. Biol..

[38] Young R.Y., Wang N., Roof W.D. (2000). Phages will out: Strategies of host cell lysis. Trends Microbiol..

[39] Cahill J., Young R. (2019). Phage lysis: Multiple genes for multiple barriers. Adv. Virus Res..

[40] Hsieh Y.J., Wanner B.L. (2010). Global regulation by the seven-component Pi signalling system. Curr. Opin. Microbiol..

[41] Keller L.M., Weber-Ban E. (2023). An emerging class of nucleic acid-sensing regulators in bacteria: WYL domain-containing proteins. Curr. Opin. Microbiol..

[42] Rojas-Pirela M., Andrade-Alviárez D., Rojas V., Kemmerling U., Cáceres A.J., Michels P.A., Concepción J.L., Quiñones W. (2020). Phosphoglycerate kinase: Structural aspects and functions, with special emphasis on the enzyme from Kinetoplastea. Open Biol..

[43] Banks R.D., Blake C.C., Evans P.R., Haser R., Rice D.W., Hardy G.W., Merrett M., Phillips A.W. (1979). Sequence, structure and activity of phosphoglycerate kinase: A possible hinge-bending enzyme. Nature.

[44] Maley F., Maley G.F. (1990). A tale of two enzymes, deoxycytidylate deaminase and thymidylate synthase. Prog. Nucleic Acid Res. Mol. Biol..

[45] Holmgren A. (1989). Thioredoxin and glutaredoxin systems. J. Biol. Chem..

[46] Stewart E.J., Åslund F., Beckwith J. (1998). Disulfide bond formation in the *Escherichia coli* cytoplasm: An in vivo role reversal for the thioredoxins. EMBO J..

[47] Rioseras B., Yagüe P., López-García M.T., Gonzalez-Quiñonez N., Binda E., Marinelli F., Manteca A. (2016). Characterization of SCO4439, a D-alanyl-D-alanine carboxypeptidase involved in spore cell wall maturation, resistance and germination in Streptomyces coelicolor. Sci. Rep..

[48] Greve J.M., Pinkham A.M., Cowan J.A. (2021). Human aspartyl (asparaginyl) hydroxylase. A multifaceted enzyme with broad intra- and extra-cellular activity. Metallomics.

[49] Bollen M., Gijsbers R., Ceulemans H., Stalmans W., Stefan C. (2000). Nucleotide pyrophosphatases/phosphodiesterases on the move. Crit. Rev. Biochem. Mol. Biol..

[50] Iyer L.M., Koonin E.V., Aravind L. (2002). Classification and evolutionary history of the single-strand annealing proteins, RecT, Redβ, ERF and RAD52. BMC Genom..

[51] Fleming J.R., Hauth F., Hartig J.S., Mayans O. (2023). Crystal structure of a GCN5-related N-acetyltransferase from Lactobacillus curiae. Acta Crystallogr. F Struct. Biol. Commun..

[52] Alekshun M.N., Levy S.B. (1999). The mar regulon: Multiple resistance to antibiotics and other toxic chemicals. Trends Microbiol..

[53] Haque M.M., Kabir M.S., Aini L.Q., Hirata H., Tsuyumu S. (2009). SlyA, a MarR family transcriptional regulator, is essential for virulence in Dickeya dadantii 3937. J. Bacteriol..

[54] Nazaret F., Alloing G., Mandon K., Frendo P. (2023). MarR family transcriptional regulators and their roles in plant-interacting bacteria. Microorganisms.

[55] Holtappels D., Alfenas-Zerbini P., Koskella B. (2023). Drivers and consequences of bacteriophage host range. FEMS Microbiol. Rev..

[56] Goldsmith D.B., Crosti G., Dwivedi B., McDaniel L.D., Varsani A., Suttle C.A., Weinbauer M.G., Sandaa R.A., Breitbart M. (2011). Development of phoH as a novel signature gene for assessing marine phage diversity. Appl. Environ. Microbiol..

[57] Wang X., Liu J., Yu Z., Jin J., Liu X., Wang G. (2016). Novel groups and unique distribution of phage phoH genes in paddy waters in northeast China. Sci. Rep..

[58] Nilsson E., Li K., Fridlund J., Šulčius S., Bunse C., Karlsson C.M., Lindh M., Lundin D., Pinhassi J., Holmfeldt K. (2019). Genomic and seasonal variations among aquatic phages infecting the Baltic Sea gammaproteobacterium *Rheinheimera* sp. strain BAL341. Appl. Environ. Microbiol..

[59] Kim S.K., Makino K.O., Amemura M.I., Shinagawa H.I., Nakata A.T. (1993). Molecular analysis of the phoH gene, belonging to the phosphate regulon in *Escherichia coli*. J. Bacteriol..

[60] Martiny A.C., Coleman M.L., Chisholm S.W. (2006). Phosphate acquisition genes in Prochlorococcus ecotypes: Evidence for genome-wide adaptation. Proc. Natl. Acad. Sci. USA.

[61] Kazakov A.E., Vassieva O., Gelfand M.S., Osterman A., Overbeek R. (2003). Bioinformatics classification and functional analysis of PhoH homologs. In Silico Biol..

[62] Maley G.F. (1978). Deoxycytidylate deaminase from T2-infected *Escherichia coli*. Methods in Enzymology.

[63] Dunigan D.D., Agarkova I.V., Esmael A., Alvarez S., Van Etten J.L. (2023). Early-Phase Drive to the Precursor Pool: Chloroviruses Dive into the Deep End of Nucleotide Metabolism. Viruses.

[64] Wikner J., Vallino J.J., Steward G.F., Smith D.C., Azam F. (1993). Nucleic acids from the host bacterium as a major source of nucleotides for three marine bacteriophages. FEMS Microbiol. Ecol..

[65] Zimmerman A.E., Howard-Varona C., Needham D.M., John S.G., Worden A.Z., Sullivan M.B., Waldbauer J.R., Coleman M.L. (2020). Metabolic and biogeochemical consequences of viral infection in aquatic ecosystems. Nat. Rev. Microbiol..

[66] Rihtman B., Bowman-Grahl S., Millard A., Corrigan R.M., Clokie M.R., Scanlan D.J. (2019). Cyanophage MazG is a pyrophosphohydrolase but unable to hydrolyse magic spot nucleotides. Environ. Microbiol. Rep..

[67] Shi K.X., Wu Y.K., Tang B.K., Zhao G.P., Lyu L.D. (2019). Housecleaning of pyrimidine nucleotide pool coordinates metabolic adaptation of nongrowing Mycobacterium tuberculosis. Emerg. Microbes Infect..

[68] Ho P., Chen Y., Biswas S., Canfield E., Abdolvahabi A., Feldman D.E. (2023). Bacteriophage antidefense genes that neutralize TIR and STING immune responses. Cell Rep..

[69] Qian P.Y., Cheng A., Wang R., Zhang R. (2022). Marine biofilms: Diversity, interactions and biofouling. Nat. Rev. Microbiol..

