# Supplementary material for: Genome Sequences of the First Phages Infecting Limnohabitans Reveal Their Global Distribution and Metabolic Potential
Source: Microorganisms. 2025 Jun 6;13(6):1324. doi: 10.3390/microorganisms13061324 (PMC12195039; doi:10.3390/microorganisms13061324)
Supplement: Supplementary file 1 [file microorganisms-13-01324-s001.zip › Supplementary Figures.pdf]

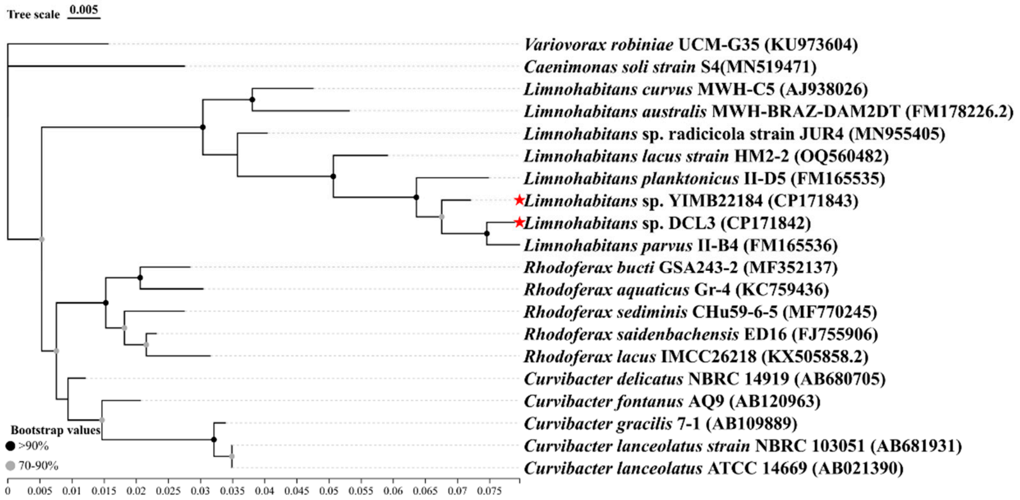

Supplementary Figure S1 The best evolutionary model for the sequence was evaluated using ModelFinder. phylogenetic tree was constructed using this model via maximum likelihood method on the IQTREE website, with a bootstrap value of 1000, and all other options were set to default. Graphical enhancement was performed via the online tool Chiplot (https://www.chiplot.online/).

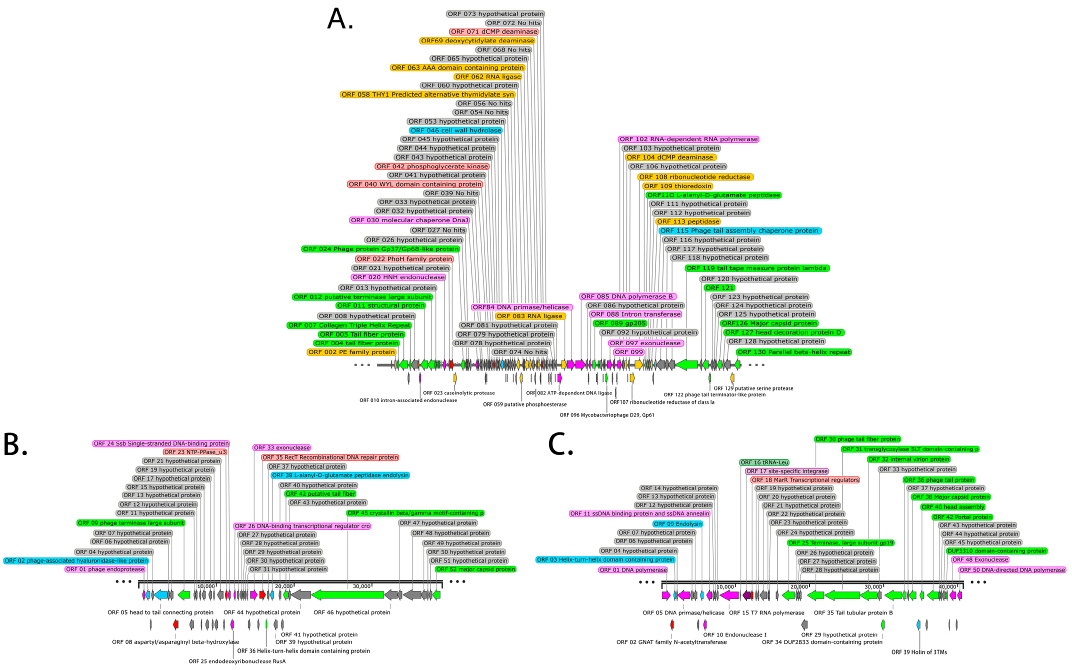

Supplementary Figure S2 Genome maps of DC31(A), DC33(B), and YIMV22061(C). Arrows represent ORFs, with different colors indicating different functions. Green: structural proteins; blue: lysis and assembly genes; pink: transcription and replication genes; red: auxiliary metabolism genes; gold: modification genes; purple: integrase gene.
